# Non-ionizing 405 nm Light as a Potential Bactericidal Technology for Platelet Safety: Evaluation of *in vitro* Bacterial Inactivation and *in vivo* Platelet Recovery in Severe Combined Immunodeficient Mice

**DOI:** 10.3389/fmed.2019.00331

**Published:** 2020-01-15

**Authors:** Michelle Maclean, Monique P. Gelderman, Sandhya Kulkarni, Rachael M. Tomb, Caitlin F. Stewart, John G. Anderson, Scott J. MacGregor, Chintamani D. Atreya

**Affiliations:** ^1^The Robertson Trust Laboratory for Electronic Sterilisation Technologies, Department of Electronic & Electrical Engineering, University of Strathclyde, Glasgow, United Kingdom; ^2^Department of Biomedical Engineering, University of Strathclyde, Glasgow, United Kingdom; ^3^Office of Blood Research and Review, Center for Biologics Evaluation and Research, Food and Drug Administration, Silver Spring, MD, United States

**Keywords:** 405 nm light, bacteria, platelets, inactivation, violet-blue light, pathogen reduction

## Abstract

Bacterial contamination of *ex vivo* stored platelets is a cause of transfusion-transmitted infection. Violet-blue 405 nm light has recently demonstrated efficacy in reducing the bacterial burden in blood plasma, and its operational benefits such as non-ionizing nature, penetrability, and non-requirement for photosensitizing agents, provide a unique opportunity to develop this treatment for *in situ* treatment of *ex vivo* stored platelets as a tool for bacterial reduction. Sealed bags of platelet concentrates, seeded with low-level *Staphylococcus aureus* contamination, were 405 nm light-treated (3–10 mWcm^−2^) up to 8 h. Antimicrobial efficacy and dose efficiency was evaluated by quantification of the post-treatment surviving bacterial contamination levels. Platelets treated with 10 mWcm^−2^ for 8 h were further evaluated for survival and recovery in severe combined immunodeficient (SCID) mice. Significant inactivation of bacteria in platelet concentrates was achieved using all irradiance levels, with 99.6–100% inactivation achieved by 8 h (*P* < 0.05). Analysis of applied dose demonstrated that lower irradiance levels generally resulted in significant decontamination at lower doses: 180 Jcm^−2^/10 mWcm^−2^ (*P* = 0.008) compared to 43.2 Jcm^−2^/3 mWcm^−2^ (*P* = 0.002). Additionally, the recovery of light-treated platelets, compared to non-treated platelets, in the murine model showed no significant differences (*P* = >0.05). This report paves the way for further comprehensive studies to test 405 nm light treatment as a bactericidal technology for stored platelets.

## Introduction

Transfusion of *ex vivo* stored platelets (PLTs) is an essential and life-saving medical intervention in bleeding, trauma, and surgery patients. Bacterially contaminated PLTs are associated with the highest risk of transfusion transmitted infection in the USA, and can lead to septic transfusion reactions (1, 2). Platelet transfusion products are particularly susceptible to contamination due to their storage at 22 ± 2°C in gas permeable bags, under constant agitation for 5–7 days post donation (3, 4).

Several methods have been implemented to reduce the risk of bacterial contamination during blood donation including donor questionnaires, skin disinfection, diversion of the initial blood donation (5) and testing samples for bacterial contaminants (5, 6). However, there is still a residual risk of platelet contamination, with ~1 in 10,000 units testing positive for contamination, 1 in 100,000 causing transfusion reactions and 1 in 500,000 causing fatal transfusion reactions (7).

According to US FDA regulations [21 CFR 606.145(a)], blood establishments and transfusion services must assure that the risk of bacterial contamination of platelets is adequately controlled using FDA approved or cleared devices, or other adequate and appropriate methods found acceptable for this purpose by FDA. Acceptable methods include bacterial testing or pathogen reduction technologies (PRTs) (8). Several PRTs are approved or under investigation worldwide. This includes Intercept system (Cerus Corporation, USA) which is an approved PRT in the USA, that makes use of amotosalen followed by UV-A (320–400 nm) irradiation for pathogen reduction (9). There are two additional systems commonly studied: Mirasol (TerumoBCT, USA), approved for use in other countries, which combines riboflavin and broad-spectrum UV light (265–370 nm); and Theraflex UV Platelets (Macopharma, France), currently under development using 254 nm UV-C light (10, 11). However, available PRTs do affect product quality *in vitro* and function *in vivo*, following transfusion (12–14). The ideal PRT would be relatively inexpensive, simple to implement and, with no or minimal effect on the quality or efficacy of the transfusion product (15).

Violet-blue light in the region of 405 nm could provide a novel alternative PRT, without the need for additional exogenous photosensitizers or extensive processing. These high-intensity visible light photons excite endogenous porphyrins within microbial cells, which causes an energy transfer and the generation of reactive oxygen species. These toxic species cause widespread oxidative damage to the pathogens, which ultimately leads to death of pathogens (16–18). This decontamination method has efficacy against a wide range of pathogenic organisms including vegetative bacteria, spore forming bacteria and fungi (17, 19, 20), with recent studies also demonstrating the potential for inactivation of viruses, protozoa, and parasites (21–23). Additionally, 405 nm light is safer than UV light wavelengths, with antimicrobial efficacy capable of being achieved at levels that are compatible with mammalian cells (16, 24–28).

A recent study (29) reported the successful reduction of bacterial contamination within human blood plasma using 405 nm light, and significantly, demonstrated the capacity for this decontamination treatment to be applied to the plasma whilst *in situ* within the transfusion bag, highlighting a potential operational advantage for this prospective PRT. Antiviral efficacy has also been indicated, with successful reduction of a model virus (calicivirus) in small volume plasma samples artificially seeded with the virus (22).

The aim of the present study was to expand on the results of these previous proof-of-concept studies, and investigate the potential of this antimicrobial technique to be applied to human platelet concentrates—which, as discussed, have a high potential for bacterial contamination due to their standard storage at room temperature conditions. The study investigated the efficacy of 405 nm light for decontamination of artificially-seeded platelet suspensions, *in situ* within transfusion bags, and also assessed the compatibility of the visible light-treated platelets for transfusion within a murine model.

## Materials and Methods

### Human Platelets

**Figure 1:**
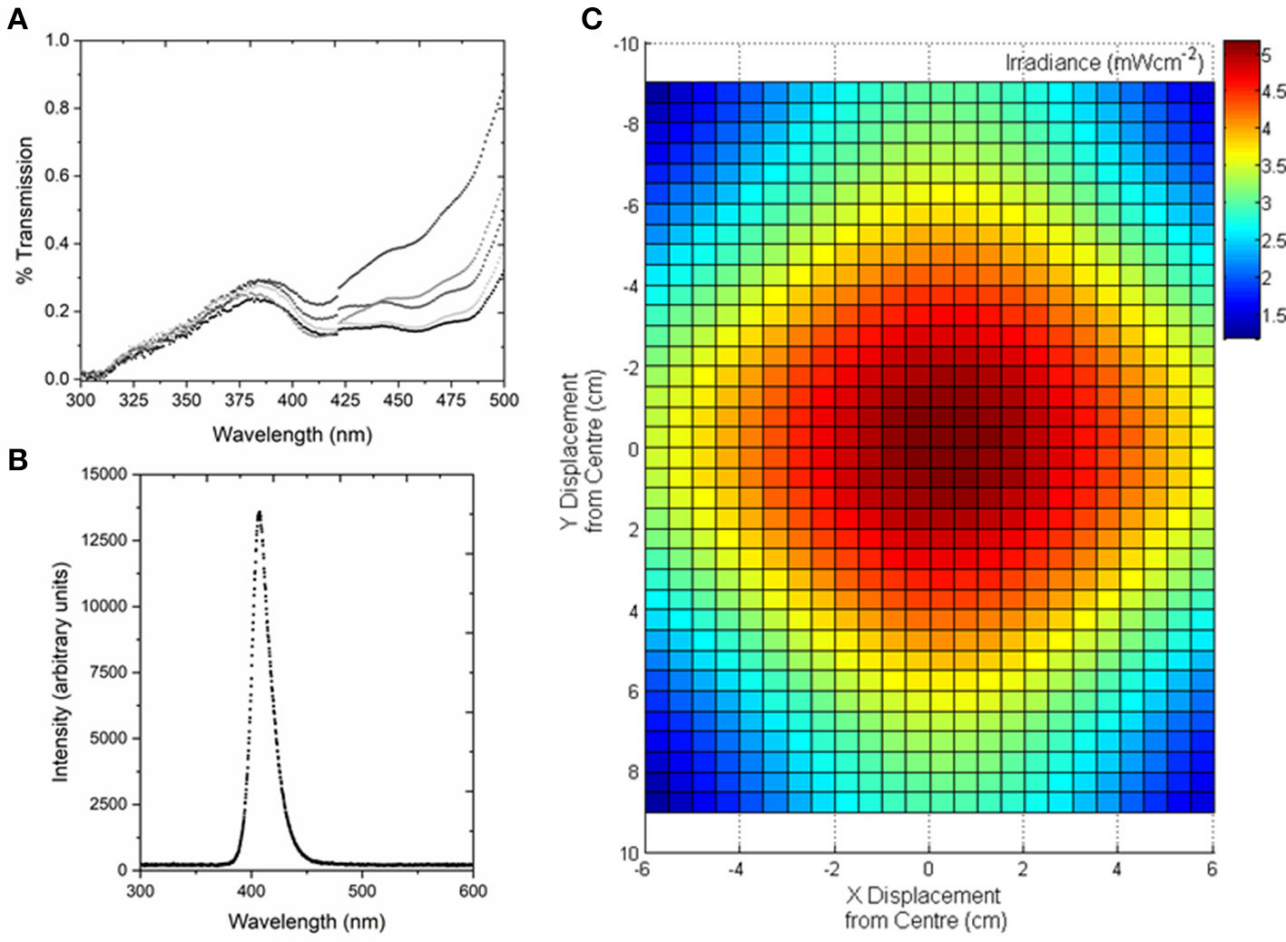
Optical characterization of human platelet concentrates, and the 405-nm light treatment system used for exposure of platelet concentrates. **(A)** Transmission of human platelet concentrates. Analysis demonstrates the slight variation in optical properties between batches (*n* = 5). Measured by UV-Vis spectrophotometry using a wavelength scan between 300 and 500 nm. **(B)** Optical emission spectrum of the 405-nm LED array, measured using a high resolution spectrometer (HR4000 Ocean Optics Inc, Germany) and SpectraSuite software (Version 2.0.151). **(C)** Model demonstrating the irradiance profile across the area of the platelet bag, with irradiance of ~5 mWcm^−2^ at the center (plotted using MATLAB R2012b software).

Human PLTs (~200 mL bag volume) were obtained from the Scottish National Blood Transfusion Service (SNBTS, UK), and the National Institutes of Health Blood Bank (Bethesda, MD, USA) and stored at 22 ± 2°C under agitation. Study involving human subjects protocol was approved by FDA Risk Involved in Human Subjects Committee (RIHSC, Exemption Approval # 11-036B) and by the University of Strathclyde Ethics Committee (UEC1011/31). Representative PLT samples were analyzed by UV-Vis spectrophotometry (Biomate 5, Thermo Spectronic) to determine the optical transmissibility of the PLTs between 300 and 500 nm, with transparency generally in the range of (>0.1– <0.3%) between 400 and 405 nm (Figure 1A).

### Bacterial Culture

*Staphylococcus aureus* NCTC 4135 (National Collection of Type Cultures, Collindale, UK) was cultured in 100 mL nutrient broth (Oxoid Ltd, UK) at 37°C under rotary conditions (120 rpm) for 18 h. Broths were centrifuged at 3939 × *g* for 10 min and the pellet re-suspended in 100 mL phosphate buffered saline (PBS), and serially diluted for seeding into human PLT bags at a concentration of 10^2^ colony-forming units per milliliter (CFU mL^−1^), in order to provide representative levels of low density contamination.

### 405-nm Light Treatment

The 405 nm light sources used were light emitting diode (LED) arrays (ENFIS PhotonStar Innovate UNO 24; PhotonStar Technologies, UK) powered by a 40 V Phillips Xitanium LED Driver (Phillips, Netherlands), with a center wavelength of 405 nm and ~10 nm FWHM (Figure 1B). The arrays were bonded to heat sinks and fans, to ensure no thermal effects on the exposed PLT samples. For PLT treatment, two 405 nm LED arrays were held in a fixed position above the horizontally-positioned platelet bag, with the optical output adjustable to provide irradiances of between 3 and 10 mWcm^−2^ at the center position of the PLT bag, taking into account a 20% reduction in irradiance as the light transmits through the bag layer. Fixed irradiances of ~3, 5, 7, and 10 mWcm^−2^ were used in this study, with an example of the optical profile of the light distribution across the transfusion bags, measured using a radiant power meter and photodiode detector (LOT-Oriel Ltd., USA), shown in Figure 1C. PLT bags were exposed to the fixed irradiances for treatment times of up to 8 h, and the treatment dose (J cm^−2^) was calculated as the product of the irradiance (W cm^−2^) multiplied by the exposure time (sec), with the irradiance value being the maximum measured at the center position of the bag.

The experimental system was held in a shaking incubator (72 rpm; 22–25°C) to allow continuous sample agitation and maintain exposure conditions. Seeded PLT bags were treated with increasing exposures of 405 nm light. Control samples, which were taken from the test samples before spiking, were held in similar conditions, but shielded from the light. Post exposure, samples were spread plated onto nutrient agar (Oxoid Ltd, UK) using standard microbiological plating methods, and incubated at 37°C for 24 h. Surviving bacteria were then enumerated and reported as % CFU survival compared to equivalent untreated control samples. Data was statistically analyzed to assess for significant differences between the % decontamination achieved using differing irradiances at the same exposure time [*P* = < 0.05, one-way ANOVA with Fishers *post-hoc* test (Minitab v18)], and to detect significant differences between treated and untreated PLT samples [*P* = < 0.05; two sample *t* test (Minitab v18)].

### SCID Mice

Six week old female immunodeficient (SCID) mice were obtained from the National Cancer Institute, Division of Cancer Treatment, NIH. They were fed *ad libitum* and allowed to acclimatize in a pathogen-free facility for a minimum of 7 days before the experiments commenced. Animal protocols were in compliance with guidelines provided by the Center of Biologics Evaluation and Research Animal Research Advisory Committee.

### Transfusion of Light-Treated Human PLTs Into Mice and Mouse Whole Blood Collection

Human PLTs (non-seeded) were exposed to 10 mWcm^−2^ light treatment for 8 h (as described above). PLTs (~1 × 10^9^ PLTs in 100 μL) were then infused into SCID mice as previously described by Bosch-Marcé et al. (30) and Piper et al. (31). Briefly, a 100 μL aliquot of concentrated human control PLTs or light-treated PLTs (Test) was injected into the right lateral tail vein with a 1-mL syringe fitted with a 27-gauge needle. At predetermined time points, 30 min, 2, 4, 6, 8, 10, and 24 h after transfusion, small samples of whole blood were collected into heparinized capillary tubes (Fisher Scientific, Pittsburgh, PA) from the left lateral tail vein using a tail vein nick technique, and the human PLT recovery in circulation was determined immediately, after incubation with a monoclonal antibody or an isotype control, using flow cytometry. The amount of PLTs recovered at 30 min after infusion was set as the 100% recovery.

### Flow Cytometric Detection of Human PLTs

Human PLTs in mouse whole blood were detected with a monoclonal anti-human CD41-FITC–conjugated antibody (Clone P2; Beckman Coulter, Brea, CA) using a flow cytometer as previously described by Bosch-Marcé et al. (30) and Piper et al. (31). Briefly, the blood samples and the antibody were incubated for 20 min at RT in the dark. After the incubation period, the samples were washed twice and centrifuged for 5 min at 1,000 × *g*, the supernatant was removed, and the cells were resuspended in PBS containing 0.1% bovine serum albumin. The samples were analyzed with a flow cytometer (FACSCalibur, BD Biosciences, San Diego, CA). The human PLTs were used to establish a gate based on forward (FSC) and side (SSC) scatter, which would detect the majority of the transfused PLTs. This gate was then used to acquire 25,000 PLT events from collected mouse whole blood. An ungated FSC vs. SSC density plot of mouse whole blood verified that the PLT population in the sample fell within the gate established from the human PLT sample. PLT recovery was defined as the percentage of anti-human CD41-FITC–positive gated events in the sample of mouse whole blood. Each mouse was its own control and therefore, for each mouse, the amount of PLTs recovered at 30 min after infusion was set as the 100% recovery for all subsequent time points. Data was analyzed [*P* > 0.05; paired *t* test (Minitab v18)] to determine any significant differences between the light-treated (*n* = 12) and non-light treated, control, PLTs (*n* = 14). Since we consistently observed negligible amounts of human PLTs at 24 h in all treatments, only data up to 10 h were analyzed for significance.

## Results

**Figure 2:**
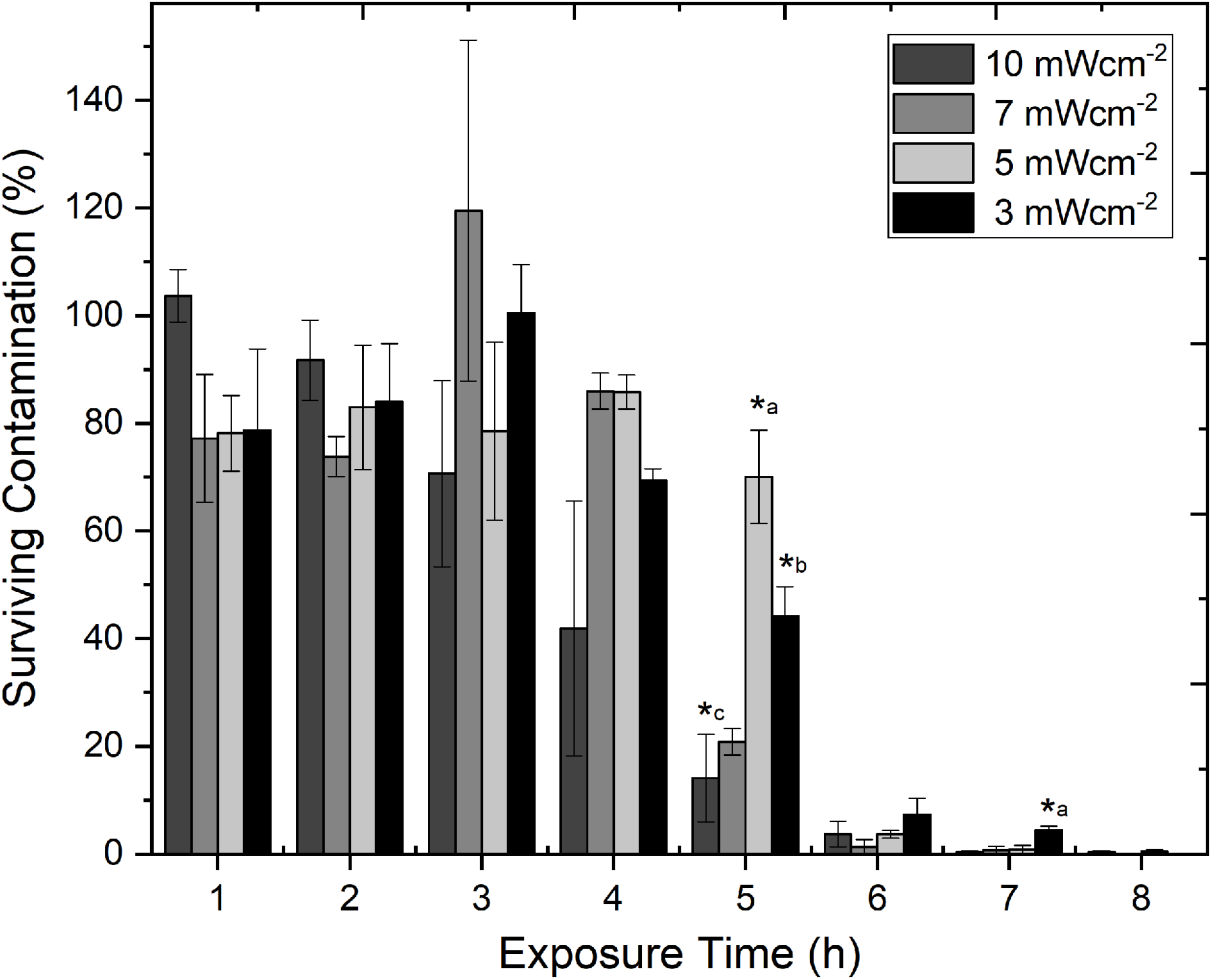
Comparison of the decontamination efficacy of varying irradiances of 405 nm light for decontamination of *ex vivo* stored human platelet concentrates, as a function of time. Platelet transfusion bags were seeded with low density contamination (10^2^ CFUml^−1^ *Staphylococcus aureus*) under constant agitation (72 rpm; 22–25°C). Data represents mean ± SEM (*n* = >3), with % survival compared to untreated (0 Jcm^−2^) platelet samples. (^*^) represent significantly different % decontamination compared to other irradiances at the same exposure time [*P* = <0.05, one-way ANOVA with Fishers *post-hoc* test (Minitab v18)]: ^*^a, significantly different to all other irradiances; ^*^b, significantly different to 5 & 10 mWcm^−2^; ^*^c, significantly different to 3 & 5 mWcm^−2^.

Figure 2 demonstrates the efficacy of 405 nm light for decontamination of PLT bags seeded with low density *S. aureus*, using the 4 fixed irradiance levels, over time. It can be seen that at all light irradiances used, successful reduction of the bacterial contamination was achieved. By 8 h exposure <0.5% contamination was detected with all irradiances used. Analysis of the inactivation kinetics of each of the 4 irradiances, demonstrated that inactivation was most effective with the highest, 10 mWcm^−2^, irradiance: contamination levels decreased in a constant downward trend from 1 h, with 0.3% remaining by 8 h. With the other 3 irradiances used, contamination levels remained consistent over the first 4 h, with the majority of the inactivation (>92.5%) achieved by 6 h exposure—similar to the 10 mWcm^−2^ treatment. Statistical analysis demonstrated that, at most exposure times (1–4, 6, and 8 h), the levels of bacterial inactivation achieved were statistically similar despite the use of different irradiance levels (*P* > 0.05). The sample-to-sample variation in PLT transmissibility (Figure 1A) is likely a contributing factor to the degree of deviation observed in some of the inactivation data, and consequently impacting statistical analysis. Regardless, significant differences in the degree of inactivation achieved at fixed time periods were detected at 5 h (*P* = 0.001), and 7 h (*P* = 0.006) (Figure 2). Bacterial contamination in non-treated control samples, showed no decrease over the 8 h period.

**Figure 3:**
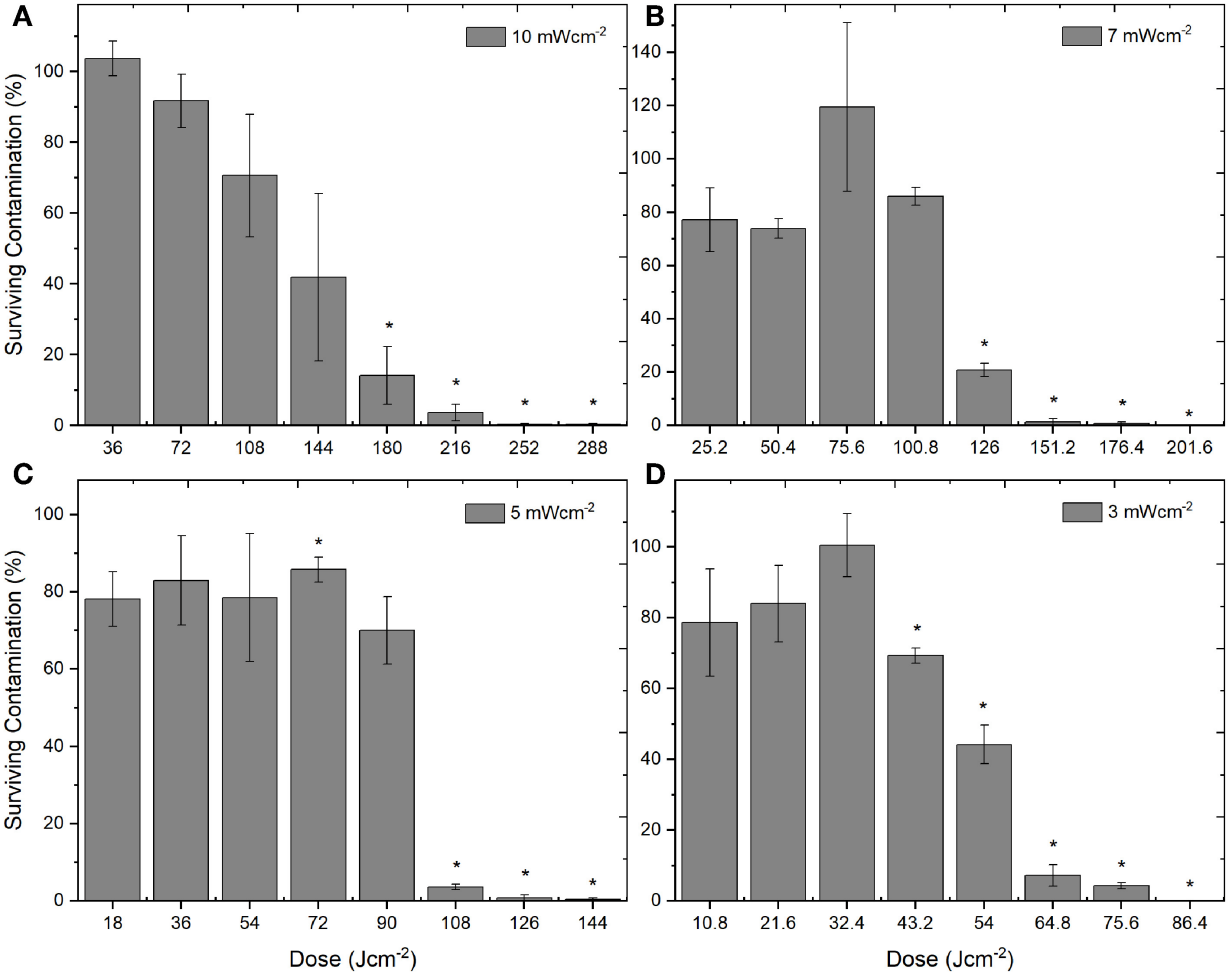
Decontamination of *ex vivo* stored human platelet suspensions using 405 nm light, as a function of dose. Platelet transfusion bags were seeded with low density contamination (10^2^ CFUml^−1^ *S. aureus*) and exposed to irradiances of approximately **(A)** 10 mWcm^−2^, **(B)** 7 mWcm^−2^, **(C)** 5 mWcm^−2^, and **(D)** 3 mWcm^−2^, under constant agitation (72 rpm; 22–25°C). Data represents mean ± SEM (*n* = >3), with % survival and significant (^*^) decontamination calculated compared to untreated (0 Jcm^−2^) samples [*P* = <0.05; paired *t* test (Minitab v18)].

Although the levels of inactivation achieved over the 8 h exposure period were comparable when using the 4 irradiance levels (3, 5, 7, 10 mWcm^−2^), the dose levels were found to be quite different. Figure 3 demonstrates the inactivation kinetics of the different irradiances as a function of dose (Jcm^−2^). The highest dose levels were used when exposing the PLTs to the highest irradiance of 10 mWcm^−2^, ranging from 36 Jcm^−2^ (1 h treatment) to 288 Jcm^−2^ (8 h treatment), with significant reductions achieved by 5 h/180 Jcm^−2^ (*P* = 0.008). As the fixed irradiance decreased, so did the applied dose levels, with irradiances of 7, 5, and 3 mWcm^−2^ giving doses ranging from 25 to 201, 18 to 144, and 10.8 to 86 Jcm^−2^, respectively. Results demonstrate that, generally, use of the lower irradiance levels, resulted in statistically significant reductions in bacterial contamination at lower applied doses: 126 Jcm^−2^ using 7 mWcm^−2^ (*P* = 0.009); 72 Jcm^−2^ using 5 mWcm^−2^ (*P* = 0.003), and 43.2 Jcm^−2^ using 3 mWcm^−2^ (*P* = 0.002).

**Figure 4:**
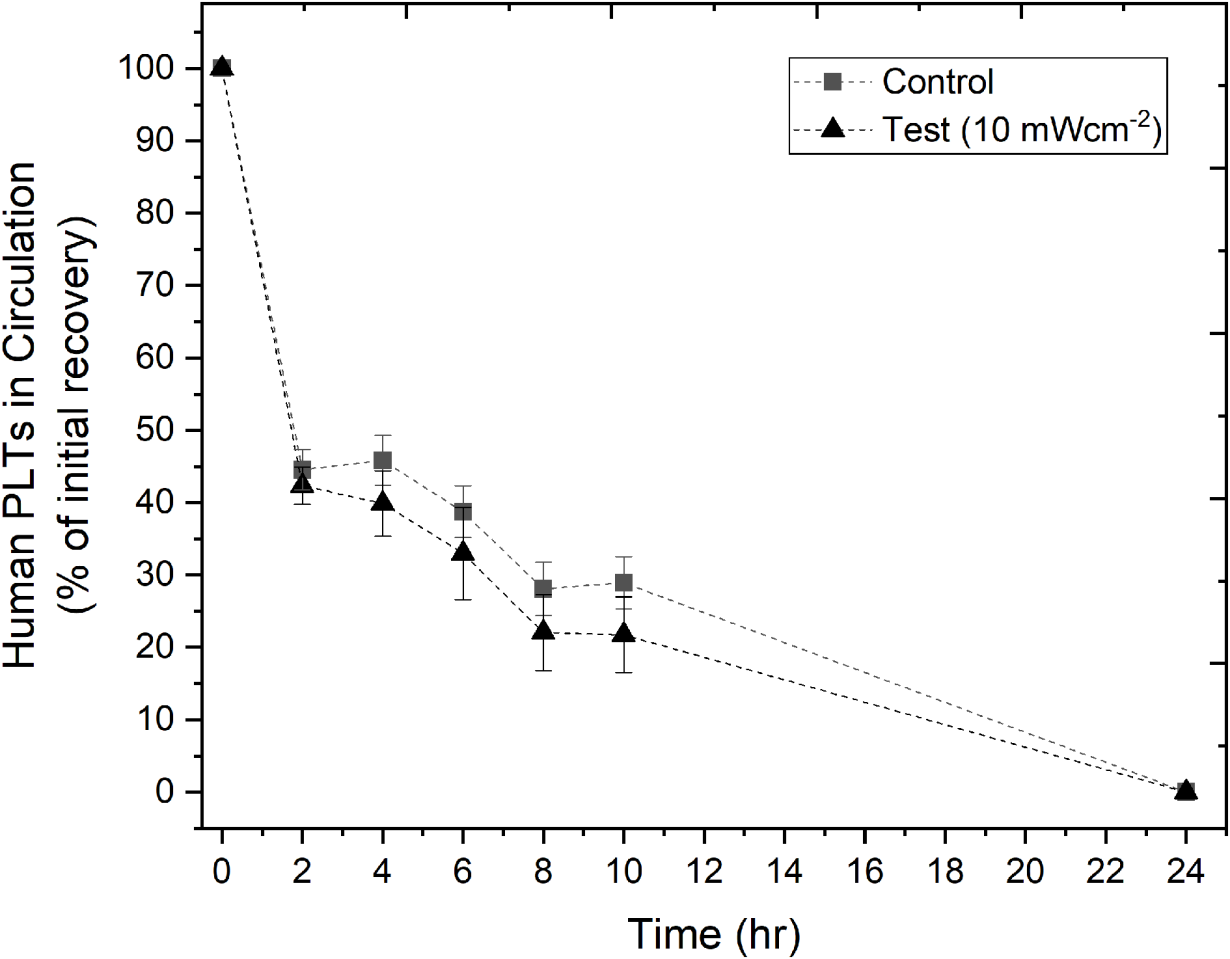
*In vivo* recovery of human platelets (PLTs) in SCID mice. PLTs were treated with 10 mWcm^−2^ 405 nm light for 8 h before transfusion into SCID mice. The % of initial recovery of non-treated (*n* = 14 ± SEM) and light-treated (*n* = 12 ± SEM) PLTs was monitored over time, up to 24 h. Negligible amounts of human PLTs were consistently observed at 24 h in all treatments, therefore only data up to 10 h were analyzed for significance. Results demonstrated no significant difference [*P* > 0.05; two sample *t* test (Minitab v18)] between the light-treated and non-light treated (control) PLTs.

To evaluate whether the treatment of human PLTs with 405 nm light had an effect on their recovery, PLTs were exposed to 10 mWcm^−2^ light for 8 h, and the *in vivo* recovery of human PLTs in SCID mice was then analyzed. In Figure 4, recovery of the human PLTs show that treatment with antimicrobial 405 nm light had minimal effect on the recovery of human PLTs in the SCID mice when compared to the control, non-treated human PLTs, with no significant differences detected at any of the tested time points (2–10 h; *P* = >0.05). Additional testing using PCs light treated for 12 h instead of 8 h demonstrated that PLT recovery was very similar to those that had been treated for 8 h (*n* = 5 for test group, *n* = 5 for control group; data not shown).

## Discussion

The results of this study has provided the proof of concept for bactericidal efficacy and cellular safety margin of 405 nm violet-blue light on *ex vivo* stored human platelets spiked with *S. aureus* whilst held within sealed transfusion bags. Use of relatively low irradiances of 405 nm light (3–10 mWcm^−2^) were shown to be effective for bactericidal activity over treatment periods of up to 8 h. Additionally, PLT concentrates which had been exposed to the maximum treatment (10 mWcm^−2^ for 8 h), also demonstrated *in vivo* recovery in a murine model similar to that of non-treated PLTs.

All four irradiances levels used in the study (3, 5, 7, and 10 mWcm^−2^) achieved good bactericidal effects, with consistent trends of inactivation rates: >92.5% reduction by 6 h, and >99% reduction by 8 h. Although there was, in the most part, no significant difference in the levels of inactivation achieved at each exposure time with the different irradiance levels, trends were observed. Use of 10 mWcm^−2^ light, resulted in the most linear inactivation kinetics, with inactivation at the lower irradiances (3, 5, 7 mWcm^−2^) displaying a more linear, and indeed upward, initial trend (up to 3–4 h) before inactivation became evident (~5 h onwards). It is possible that this difference in kinetics may be due to the fact that at the lower irradiances, the low density bacterial contaminants are slower to be affected by the light treatment, and are able to persist and, to some extent, potentially replicate, in these early stages, with room temperature storage providing adequate conditions for growth.

Interestingly, the inactivation kinetics observed for platelets are very similar to those shown for human plasma in a previous study by the authors (29). The study demonstrated use of 5 mWcm^−2^ 405 nm light for treatment of pre-bagged human blood plasma, with contamination levels remaining steady up till a treatment dose of 90 Jcm^−2^ (as shown with the same irradiance in the present study), followed by significant decontamination being achieved with doses ≥108 Jcm^−2^. This similarity in inactivation rates between platelets and plasma is of great interest, particularly when looking at their comparative optical transmissibility. Analysis of human PLT samples (*n* = 5) demonstrated low transmissibility of 405 nm light, with variation in transmission of between 0.1 and 0.2%. The transmissibility of plasma tends to be slightly greater (1–2%) in the region of 405 nm (29). With this low transmissibility, and also when observing the non-uniform optical profile over the area of the PLT bags, continuous agitation of the bags during light treatment is important to ensure uniform mixing of the PLT contaminants, and prevents shading of contaminants at the lower depths of the PLT concentrates.

Analysis of the applied optical doses used for decontamination highlighted that although successful inactivation was achieved using each of the 4 irradiance levels, the doses required to achieve this inactivation varied greatly. Whilst the levels of inactivation achieved at each of the treatment times were generally comparable despite the different light irradiances used (3, 5, 7, 10 mWcm^−2^), the applied dose levels are quite different. Using 6 h exposure as an example (Figure 3), it can be seen that the doses required to achieve the same >92.5% reduction vary depending on the irradiance level used—ranging from 64.8 Jcm^−2^ (when using 3 mWcm^−2^) up to 216 Jcm^−2^ (using 10 mWcm^−2^). When this is considered in relation to the antimicrobial mechanism of action, it is likely that this improved efficiency at lower irradiance levels is due to the ratio of photons to intracellular porphyrins: there will be a threshold of the number of photons that can actively be involved in photoexcitation of the endogenous intracellular porphyrins at any given time, and above this there is likely a point at which the absorption of more photons will have little effect on the inactivation mechanism already in progress (32). For practical application, it will be important to evaluate any potential implications of these excess photons, in order to ensure that there is no detrimental effect to the PLT integrity due to concurrent photoexcitation of any other photo-sensitive components within the PLT concentrates themselves, in addition to assessing electrical and optical efficiencies.

As a proof-of-concept, this study used *Staphylococcus aureus* as the model organism for antimicrobial testing. Previous studies have demonstrated the bactericidal efficacy of 405 nm light for a limited selection of Gram-positive (*S. aureus* and *Staphylococcus epidermidis*) and Gram-negative bacteria (*Escherichia coli*), and also viruses (feline calicivirus) within blood plasma (22, 29). Based on these studies, we believe, that similar inactivation efficacy would be achieved in PLT concentrates, particularly given the similar inactivation kinetics observed for *S. aureus* between this study and the previous work by the authors (29). However, as the susceptibility of a bacterial species to a specific photo-inactivation method can vary between strains, future work will employ a panel of different bacterial species and strains that are recommended by the World Health Organization (WHO) in order to validate this technology for PLT safety from blood borne bacteria and other microbes. In addition to this, expanding testing to include antimicrobial evaluation against both high and low concentrations of bacterial contamination will be important to evaluate inactivation kinetics, and demonstrate the potential for maintaining sterility, respectively, to the end of product's shelf life.

While with current PRTs, the germicidal efficiency of photodynamic inactivation using UV-light coupled with a photosensitizer is higher than that of 405 nm violet-blue light alone, the use of 405 nm light does have a number of envisaged operational and safety advantages. Violet-blue light utilizes non-ionizing light, and this is of practical interest as there are some concerns that ionizing UV light can potentially lower platelet recovery and decrease the recirculation of pathogen inactivated-treated platelets (9, 33). It is thought that, at some levels, UV light can damage platelet mRNA and ribosomal machinery that is required for protein synthesis and affect platelet proteins (particularly fibrinogen receptors), as well as application of fast dose rates producing high temperatures that can hinder platelet function (33). In the case of 405 nm light treatment, further biochemical and molecular evaluations of the platelet integrity are required in order to gain a broader understanding of the cellular interactions with photons of this wavelength at a range of irradiances and exposure times, however the proof of concept demonstrated in this study in a murine model, that the treatment conditions used (10 mWcm^−2^ for 8 h/288 Jcm^−2^) were not detrimental to the platelets compared to that of non-treated platelets, demonstrates its potential for bacterial inactivation of PLT suspensions.

The observation that 405 nm light can *in situ* inactivate bacteria in bagged PLTs, without the requirement for additional chemical additives, will have significant operational advantages in terms of reducing processing requirements, which consequently would reduce opportunities for bacterial contamination to be introduced, and potentially reduce loss of platelets due to surface binding to the containers during processing (9, 33). Technologies utilizing UV-light typically require the PLTs to be transferred to UV-transmissible bags prior to treatment, and the photosensitive chemical additive to be removed following illumination, resulting in further processing and minimal residues within the PLT concentrates (9, 33). Violet-blue light treatment would not require these processing stages.

Currently in the U.S., platelets can be stored for 5–7 days however after accounting for time required for viral screening, bacterial detection, and shipping to transfusion location, the effective shelf life is typically shorter (34). Therefore, future investigations should focus on the potential for pathogen reduction treatment using 405 nm light to improve the safety and shelf life of PLT concentrates, either through low irradiance continuous exposure throughout the storage period, or as pre-storage PRT. This could reduce the volume of wasted platelet donations: currently 4–16% of collected platelets are wasted because they do not find a recipient within their limited shelf-life (34).

Overall, this study has successfully provided a proof of concept that 405 nm light has the potential to be considered as a technology for bacterial reduction in stored human blood platelets. Both this study, and the previous work on the potential of 405 nm light for decontamination of blood plasma (29), have utilized small scale systems for product treatment, and there are a variety of both antimicrobial and hematological investigations, and technological challenges, which will need to be investigated in order to fully evaluate the compatibility and practicality of this antimicrobial technology. However, the penetrability and non-ionizing nature of 405 nm light, and the non-requirement for photosensitizing agents, provide unique benefits for 405 nm violet-blue light as a technology for *in situ* treatment of *ex vivo* stored platelets.

## Data Availability Statement

The datasets generated for this study are available on request to the corresponding author.

## Ethics Statement

The animal study was reviewed and approved by Center for Biologics Evaluation and Research, US-FDA Institutional Animal Care and Use Committee, accredited by AAALAC International.

## Author Contributions

MM, MG, JA, SM, and CA contributed to the conception and design of the study. MM, MG, SK, and CA carried out experiments and analyzed data. MM, MG, CA, RT, and CS wrote sections of the manuscript. All authors contributed to manuscript revision, read and approved the submitted version.

### Conflict of Interest

CA, MM, JA, and SM have filed a joint US device patent application. The remaining authors declare that the research was conducted in the absence of any commercial or financial relationships that could be construed as a potential conflict of interest.
